# The scenario of self-medication practices during the covid-19 pandemic; a systematic review

**DOI:** 10.1016/j.amsu.2022.104482

**Published:** 2022-08-27

**Authors:** Abhigan Babu Shrestha, Manjil Aryal, Junu Rana Magar, Sajina Shrestha, Labiba Hossainy, Fahmida Hoque Rimti

**Affiliations:** a M Abdur Rahim Medical College, Rajshahi University, Dinajpur, Bangladesh; b Central Institute of Science and Technology, Pokhara University, Kathmandu, Nepal; c KIST Medical College, Imadol, Patan, Nepal; d Department of Pediatrics, Shaheed Ziaur Rahman Medical College Hospital, Bogra, Bangladesh; e Chittagong Medical College, Chittagong, Bangladesh

**Keywords:** COVID-19, Drugs, Pandemic, Review, Self-medication

## Abstract

**Background:**

Self-medication association with an ongoing pandemic is evident in the studies conducted throughout the world. To summarize the findings of previous papers, we carried out a systematic review to observe the current scenario of self-medication during COVID-19.

**Methodology:**

Scopus, Embase, Web of Science, PubMed, MedRxiv preprints, SciELO Preprints, google, and google scholar were searched using keywords related to the topic. Studies reporting original data and assessing the self-medication practices during Covid-19 were included.

**Results:**

A total of 660 papers were collected and 14 cross-sectional studies among them were finalized from 12 different countries after apposite screening processes. Our study measured that during the COVID-19 pandemic, there was a 44.786% prevalence of self-medication. Analgesics, antibiotics, and nutritional supplements were commonly practiced drugs. Pharmacy and hospital outlets were the main sources of the drugs. Fever, sore throat, body ache (muscle pain), and flu or cough were among the most frequently recorded illnesses; treatment and prevention of COVID-19 were the main culprit behind self-medication. During COVID-19, the major factors associated with self-medication were fear, anxiety, and perception regarding COVID-19. Thus, in this pandemic, fear, anxiety, and rumors regarding immunity boosters, nutritional supplements, financial burden, and easy accessibility to even non-OTC drugs; all have their fair share in self-medication practices.

**Conclusion:**

As there was heterogeneity regarding COVID-19 and self-medication found among the assessed studies, educating general people about safe self-medication practices, hazards of superfluous drug usage, and provision of an affordable quality-health system should become a priority, especially in low and middle-income countries.

## Highlights

• Self-medication is the usage of drugs for the treatment of self-diagnosed diseases. The COVID-19 has influenced the pattern of self-medication and has brought a new trend in the society.
• In our finding, the prevalence of self-medication was found to be 44.78% amidst pandemic. Analgesics, antibiotics and nutritional supplements were commonly practiced.
• Pharmacy and hospitals were the major sources of the drugs. The major factors associated with self-medication were fear, anxiety, and perception regarding COVID-19.

## 1 Introduction

Since the very first outbreak of Coronavirus in the provision of Wuhan, China, the COVID-19 pandemic has completely changed people's way of living in most countries [1]. All around the world, the rampant spread of coronavirus has created chaos and havoc, not to mention the continuous struggle of governments and health care workers to treat infected people and stop the spreading of infection.

Countries around the world have imposed lockdown according to the need of controlling the infection. However, many small clinics and treatment facilities have closed during COVID-19, and the fear of contracting the virus aided the deprivation of time-sensitive medical advice or assistance when needed by the common people. It is an encouraging factor for self-medication [2].

World Health Organization narrates that the usage of drugs for the treatment of self-diagnosed diseases and in some cases, self-medication prescribed by a doctor for treating chronic diseases, recurrent diseases, or symptoms are known as self-medication [3]. Whereas, incongruous self-medication is when people irresponsibly consume medication [4]. Usage of old drugs, drugs prescribed for other symptoms or diseases, usage of drugs without prescription from a doctor, medication sharing between friends and family, and usage of expired drugs are all included in this type of self-medication [5].

Lockdown measures, limited access to hospitals and health centers due to COVID-19, and fear of acquiring infection from healthcare center employees or patients are amongst the most common contributing factors that encourage self-medication [2]. Self-medication practices ought to be monitored properly, especially among the low and middle-income countries which are currently facing an economic crisis due to COVID-19 and often has low standards of education along with insufficient healthcare facilities [6].

Incongruous self-medication could pose many threats to its users such as side effects of improper medication, misdiagnosis, delay in seeking professional advice, improper selection of therapy, taking medications with unforeseen complications, taking wrong doses of medication, dependence risk, and so on [7]. According to various studies, self-medication has a prevalence rate of 32.5–81.5%, making it a common exercise worldwide [8]. The situation has also been provoked by the rise of misinformation about self-medication in the social media sphere which leads to panic and confusion and further increases the rate of self-medication including home remedies that do not possess established safety or efficacy [9].

Several drugs have been presented as prospective candidates for the COVID-19 pandemic as it has progressed [10], with little to no help provided for the patients by the majority [11,12]. Some are even responsible for causing harm [13]. Findings from small investigations or in vitro studies presented a drug named hydroxychloroquine which garnered a lot of attention at first [14] but later, RCT (Randomized Controlled Trial) investigations among hospitalized patients (Recovery study and Solidarity Trials) found no evidence to support the therapeutic effects of the drug, compared to standard treatment [12,15]. Azithromycin is another drug that is on this list [[16], [17], [18], [19]]. Additional drugs such as ivermectin [[20], [21], [22]] or vitamin supplements [23,24] are still undergoing well-designed trials to evaluate their efficacy against COVID-19.

Despite these results, self-medication is preferred by many people due to a lack of access to healthcare, misinformation, and mostly the fear of getting infected by COVID-19. According to the World Health Organization (WHO), unintended effects could include adverse events, needless expenses, reluctance in seeking out professional help, drug interactions, and symptom masking [[25], [26], [27]]. The methods used to quantify self-medication [5], demographic variables [4,5,28], and across different nations and circumstances [[29], [30], [31]] all influence the prevalence of self-medication.

Previous research has looked into the prevalence and characteristics of self-medication during this pandemic, and the usage of a variety of pharmaceuticals, herbal remedies, and supplements for the treatment and prevention of the COVID-19 virus [32]. But, we were unable to find many review papers to summarize their findings as of writing this paper. Hence, an updated and robust review was warranted to determine the scenario of self-medication during the COVID-19 period.

The primary objective of our systematic review was to observe the current scenario of self-medication regarding the management or prevention of COVID-19. The secondary objective was to assess the common illnesses for this practice, reasons for self-medication, the source of this medication obtainment, the type of drug taken, and any adverse events that occurred as a result of it.

## 2 Methods

To report this systematic review, The Preferred Reporting Items for Systematic Reviews and Meta-Analyses (PRISMA) criteria were used. (Supplementary Table S1) [33]. This review has been registered on PROSPERO (Registration Number: CRD42022312175) and researchregistry.com with UIN: reviewregistry1414.

### 2.1 Eligibility criteria

We included studies that fulfilled all of the following criteria: 1) cross-sectional studies either published in scientific journals or with full text available, including preprints 2) self-medication practice in the general population regardless of age and location 3) Published within the time frame of January 2020 to January 2022.

A study was considered to have evaluated self-medication when the following criteria were met: 1) A study stated to have evaluated self-medication during COVID-19 (with a different definition), or 2) A study evaluated the usage of drugs that were not prescribed by healthcare professionals during COVID-19.

Only cross-sectional studies were sought to reduce the risk of bias due to study design and preprints were included to maintain transparency, greater coverage, and visibility of the unpublished data. In addition, case reports and series, literature reviews, conference abstracts, and editorials were debarred.

### 2.2 Search strategy and study selection

**Fig. 1:**
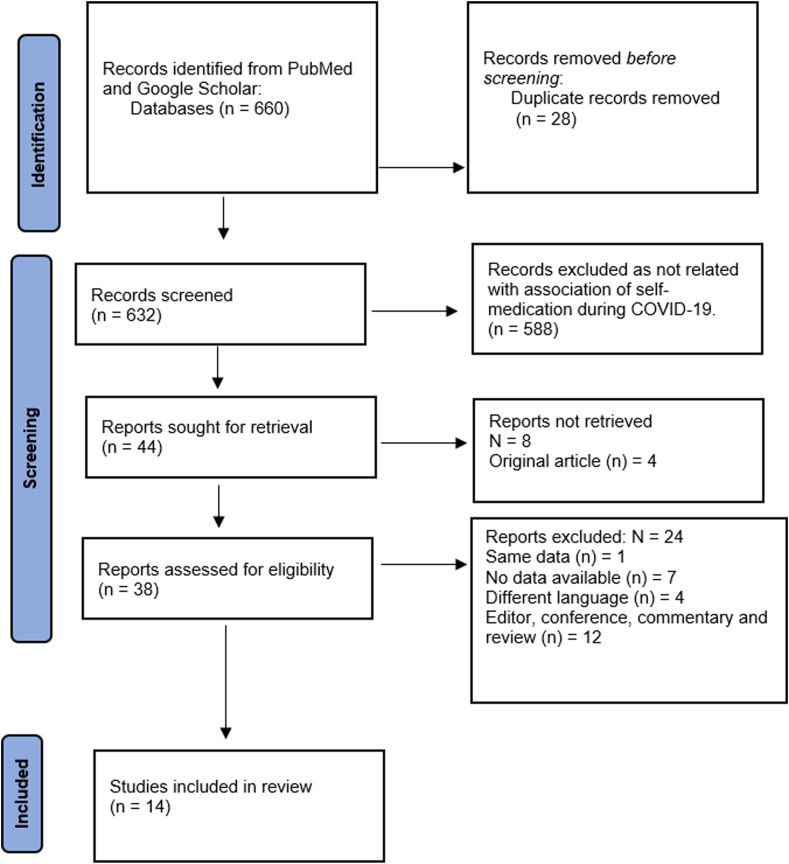
PRISMA flowchart showing selection stratergy.

Using several phrases concerning; self-medication and ‘COVID-19’ search techniques were generated (S1 Table). Scopus, Web of Science, Embase, PubMed, MedRxiv preprints, SciELO Preprints, google, and google scholar were searched from January 2020 to January 2022. No language constraint was imposed. The search strategy is depicted using the PRISMA flowchart, Fig. 1.

### 2.3 Data extraction

Studies were initially extracted from electronic databases and exported to the MENDELEY reference manager software version 1.19.8. Then the duplicate articles were first removed using MENDELEY followed by manual removal. Based on the inclusion/exclusion criteria, two authors (A.B.S and S.S) screened the articles with title and abstract. Then systematic screening for the full text was done to retrieve the final papers. Any discrepancies between the two authors were discussed with the third author (JRM).

Data were extracted on Microsoft Excel version 2016 (Microsoft Corp., Redmond, WA, USA) with the following information: author, publication year, country, study subjects, sample size, recall period, the prevalence and characteristics of self-medication practices during the COVID-19 period, common illness, drugs used, reasons of practice, factors associated, sources of drugs and adverse reaction. For similar data, studies with larger samples were extracted.

### 2.4 Assessment of methodological quality

The quality assessment was conducted by two authors (A.B.S and S.S) independently, using the risk of bias tool proposed by the Joanna Briggs Institute for critical appraisal of cross-sectional studies. The studies were scored from 0 to 8 (>5 = high quality, 4–5 = medium quality and <4 = low quality). The second author (M.A) assisted to solve the discrepancies. S2 Appendix. The AMSTAR 2 checklist was also completed to evaluate the study quality [34].

## 3 Results

### 3.1 Study characteristics

The 14 studies conducted in different countries throughout the world differed considerably in the time frame, subjects, and sample size [[34], [35], [36], [37], [38], [39], [40], [41], [42], [43], [44], [45], [46], [47]]. Though most of them focused on self-medication practices relative to COVID-19, two studies among them, namely, (i) Zhang et al., Australia [44] and (ii) Wegbom et al.*,* Nigeria [43] focused mainly on antibiotics usage during COVID-19. Eleven studies monitored self-medication practices among community people, one study among undergraduate medical students, and one study among hospitalized adults with COVID-19 amid this pandemic. Among 12 studies monitoring community people, 5 mentioned them in the general population while, one of each monitored the elderly community, adult and teenage community, educated adult community, literate smartphone accessed community, and urban community respectively. One study was conducted among frontline workers (Healthcare, Air transport, Police, Road transport, and Informal sectors). All studies were conducted by cross-sectional sampling technique in 12 different countries with sample sizes varying from 132 to 3792. The majority of the study were conducted in 2021 and five in 2020. Table 1 provides individual study characteristics descriptions with details.

**Table 1:** Characteristics of included studies such as year, country, subject, sample size and study design.

Author	Year	Country	Subject	Sample	Study Design
Sadio et al.	2021	Togo	Healthcare, Air transport, Police, Road transport and Informal sectors	955	cs
Dare et al.	2021	Uganda	Community	273	cs
Rathi et al.	2021	India	Community	1704	cs
Chopra et al.	2021	India	Community	1100	cs
Azhar et al.	2021	Pakistan	Community	290	cs
Quispe-Canari et al.	2020	Peru	Community	3792	cs
Heshmatifar et al.	2021	Iran	Elderly Community	342	cs
Nasir et al.	2020	Bangladesh	Literate adults	626	cs
Rafiq et al.	2021	Pakistan	Adult and teenage Community	920	cs
Wegbom et al.	2021	Nigeria	Literate smartphones accessed community	461	cs
Zhang et al.	2021	Australia	Urban Community	2217	cs
Minan-Tapia et al.	2020	Peru	Undergraduate students of health-related careers	718	cs
Zavala-Flores et al.	2020	Peru	Hospitalized community	132	cs
Mansuri et al.	2020	Kingdom of Saudi Arabia	Community	388	cs

cs, cross-sectional study.

### 3.2 Study quality

The risk of bias tool proposed by the Joanna Briggs Institute for critical appraisal of cross-sectional studies was employed to discern the study quality [48]. Among 14 included studies, 11 studies were of higher quality (score >5) and 3 studies were of medium quality (score 4–5). S3 Appendix. The AMSTAR 2 checklist was also completed to evaluate the study quality [34].

### 3.3 Definition of self-medication

Every study included assessed self-medication through self-reporting. Only one study [47] indicated the questions that were used for self-medication assessment, while five other studies report having inquired about drug use without prescription [31,41,43,45,46], and two of the studies [35,39] provided nothing specific about how they examined self-medication in their study.

### 3.4 Self-medication prevalence during COVID-19

All 14 studies evaluated the prevalence of self-medication during the COVID-19 pandemic. The mean prevalence rate of self-medication during the COVID-19 pandemic from around the world was calculated to be 44.78%. Only 7 studies specified recall periods ranging from 2 weeks/14 days to 6 months. Two studies gave the recall period of the duration of the COVID-19 pandemic which at that was estimated to be around two to three months. No similarities could be assessed between the recall period and self-medication prevalence rates.Table 2.

**Table 2:** Prevalence rate of self-medication and the recall period during COVID-19 pandemic from year 2020–2021.

Author	Year	Recall period	Prevalence rate during COVID-19
Sadio et al.	2021	2 weeks	34.20%
Dare et al.	2021	NR	57%
Rathi et al.	2021	NR	45%
Chopra et al.	2021	NR	25%
Azhar et al.	2021	NR	53%
Quispe-Canari et al.	2020	NR	33.30%
Heshmatifar et al.	2021	3 months	56.40%
Nasir et al.	2020	4 months	88.33%
Rafiq et al.	2021	6 months	67.3% adults; 46.9% teenagers
Wegbom et al.	2021	2 months	41%
Zhang et al.	2021	15 days	19.50%
Minan-Tapia et al.	2020	3 months	51.3%
Zavala-Flores et al.	2020	Since Covid-10 Pandemic	33.9%
Mansuri et al.	2020	Since Covid-19 Pandemic	35.1%

NR, Not Reported.

### 3.5 Common illnesses for self-medication and reasons for self-medication practices

Only 8 studies mentioned the common illness. Among them, fever, sore throat, body ache (muscle pain), and flu or cough were most frequent. Other illnesses included diarrhea, headache, lethargy, allergies, dental, and anxiety problems. The main reason for practicing self-medication was for the management and prevention of COVID-19. Similarly, previous habits, easy access, financial compliance, and fear of being infected by coronavirus were among other main reasons to practice self-medication. Table 3.

**Table 3:** Common illness, reasons and factors associated with self-medication during the COVID-19 pandemic.

Author	Common illness for self-medication	Reasons for self-medication	Factors associated with self-medication
Sadio et al.	NR	NR	Education level: secondary (P = 0.043) University (P < 0.01) Female (P < 0.01) Health sector worker (P = 0.01)
Dare et al.	NR	Fear of visiting hospital Fear of diagnosing COVID-19 positive Fear of visitng hospitals Affordability of self-medication For convenience	NR
Rathi et al.	NR	NR	NR
Chopra et al.	Headache, pain abdomen, Anxiety disorders, insomnia	NR	Gender, Marital status, Family setup and Self rating Anxiety Scale (p < 0.01)
Azhar et al.	Fever 50%, dry-cough 43.3%, throat pain 26.7%, body ache 34.7%, loss of smell and taste 20.7%, diarrhea 14.7%	Self-habit 10.7% Fear of getting coronavirus 16.3% Unavailability of doctor 27.8% Financial issue 1.1% less efficacy of doctor prescribed medicine 0.7% Bad experience with doctor 1.9%	Azithromycin (p < 0.01) Hydroxycholoquinone (p = 0.008) Ivermectin (p < 0.01) Herbal medicine (Sana Makhi) (p = 0.036)
Quispe-Canari et al.	Respiratory symptoms: Fever, muscle pain, cough, fatigue, sneezing, sore throat, nasal congestion, breathing difficulty, headache	Covid-19 positive Covid-19 symptoms Covid-19 preventive Cold or flu No symptoms Consume it regularly	Old age (P = 0.043) Employed (P = 0.028) Geographic; acetaminophen; Andes region(P = 0.001) and Rainforest(P = 0.012)
Heshmatifar et al.	joint and muscle pain 43.4% neurological diseases 42% pseudo-corona symptoms 41.9% weakness and lethargy 31% GIT 20% CVS disorders 12%	COVID-19 prevention 52.3% Avoidance of going out 51.5% Inability to afford payment or visitation 27.4% prior experience with the drug 25.3% Media, Pharmacy or other people's recommendation 23.3% Lack of coverage by insurance 17% symptoms of mild nature 16.5% Easy access to medication 15.5% Not believing in common methods of treatment 10.8% Not going near doctor's clinics or offices 7.2% Insufficient knowledge about the drug's arbitrary effects 6.2% Lack of time for physician visit 5.1%	Education and Insurance coverage(P < 0.05)
Nasir et al.	Fever 37.61%, throat pain28.7%, dry-cough 14.20%, loss of smell 9.21%, loss of taste 3.45% body ache 4.99%, diarrhea 1.72% NO symptom 16.77%	Doctor's advice 28.59% media or internet 27.15% dispensary or pharmacy 24.44% family or friends 19.8%	NR
Rafiq et al.	Fever flu or cough skin allergy, rhinitis, diarrhea	NR	Fear of possible COVID-19 infections in hospitals and interactions with pathogen carriers (P < 0.01)
Wegbom et al.	NR	COVID-19 treatment and prevention 41% Illness of emergency nature 49.1% delays in receiving hospital service 28.1% healthcare facility distance 23% The pharmacy being comparatively closer 21% non-availability of medicine in a healthcare facility 19.3% Healthcare facility costs 15.3%	Covid-19 treatment perception and perceived treatment(p < 0.005)
Zhang et al.	NR	Protection against COVID-19 Experience in using antibiotics for cold/flu 35.6% take leftover antibiotics 23.2% acquire from friends/family 17.9% Easily acquire from doctor 47.1% Misunderstand for flu/cold treatment OR = 1.6	Age(P = 0.89) Gender (P = 0.010) Education (P = 0.001) antibiotic takers due to psychological distress of covid-19 (p < 0.01)
Miñan-Tapia et al.		Perception of symptoms not being urgent enough to visit a healthcare professional or a facility (64.3%) Referring to family/friends who are not medical professionals(34.9%) Financial situation or over the counter prescription (34.9%)	
Zavala-Flores et al.	COVID-19 related symptoms	NR	NR
Mansuri et al.	Fever	Protection against Fever or COVID-19 symptoms	NR

NR, Not Reported.

### 3.6 Factors associated with the practice of self-medication in the pandemic

Only a few of the studies reviewed the association between sociodemographic, anthropological, and pandemic-based data with self-medication practices. Table 3 correlates factors like age, gender, educational status, occupational status, the field of occupation, geography, fear, anxiety, and perception regarding the COVID-19 pandemic with different levels of self-medication practices among study subjects. (Table-3)

### 3.7 Drugs used for self-medication practices, their sources, and adverse effects

Among 14 studies, only 11 of them have collected and assessed information regarding either individual drugs or their classes. The most used were different NSAIDs and Analgesics like acetaminophen, aspirin, ibuprofen, etc. followed by nutritional supplements mainly vitamin C and calcium tablets, antibiotics, and anti-allergic, and anti-malarial drugs. These drugs were mainly obtained from pharmacies and hospitals using previous prescriptions, recommendations of friends/family, or pharmacists. Many studies advocate people in developing and under-developed countries follow herbals and faith-based medicine either solely or in combination with newer medicine. Only 3 studies mentioned the side effects, of which, more commonly gastrointestinal problems followed by fever and headache. Table 4.

**Table 4:** Sources, Adverse effects and drugs used.

Author	Sources	Adverse effect	Drugs
Onchonga et al.	Pharmacy stores 89.5% Primary health facilities 10.5%	adverse drug reaction after covid22.4% while no adverse reaction before COVID-19	NR
Sadio et al.	NR	NR	Chloroquine 2% Azithromycin 1.2% Traditional medicine 10.2% Vitamin C 27.6%
Dare et al.	NR	NR	NR
Rathi et al.	NR	NR	Antacid 16%, Analgesic 13% Antitussive 10%, Vitamins 9% Antibiotics 8%, Antiseptics 8%, Anti migraine 8%, Anti inflammatory 6% Gastrointestinal 5%, Dermatological 5%
Chopra et al.	NR	NR	NSAIDS 36% Antacid 18% Antihistamine 15% Benzodiazipines 14% Antimicrobials 6% Multivitamins 7%
Azhar et al.	Prescription written for family 19.6%, for friend 3.3% Pharmacy/dispensary 19.6%	Adverse effects 5.6%	Allopathic (18.5%) Azithromycin 21.5% Hydrochloroquinone 2.6% Ivermectin 3.3% Aspirin 5.2% Dexamethasone cough syrup 16.7% Paracetamol 43.3% Vitamin C 27% Vitamin D 18.5% Calcium 14.8% Herbal medicine (5.6%) Both (28.9%)
Quispe-Canari et al.	NR	NR	Acetaminophen 27.0% antiretroviral 1.6% Azithromycin 4.8% Hydroxychloroquine 0.7% Ibuprofen 7.4% Penicillin 2.3%
Heshmatifar et al.	NR	NR	Antibiotics 27.1% Anti-cold 44% Cardiac drug 17% Gastrointestinal drug 25.9% Pain killers 52% Vitamins and supplements 47% Sedative 42.6%
Nasir et al.	NR	NR	Azithromycin 54.15% Calcium supp. 41.37% Doxycycline 40.25% Hydroxychlorquine 20.44% Ivermectin 77.15% Montelukast 43.13%
Rafiq et al.	NR	NR	Analgesics antibiotics anti-allergy cough syrups
Wegbom et al.	Pharmacy 73.9% patent medicine vendor 23.6% hospital 7.6% hawkers 4.5% faith-based outlets and herbalists 2.1%	NR	Vitamin c and multivitamins 51.8% anti-malarial other than Hydroxychloroquine and chloroquine 47.1% Amoxicillin 24.9% Ciprofloxacin 14.6% Herbal products 10.2% Metronidazole 8.5% Erythromycin 5.3% Hydroxychloroquine and Chloroquine 3.2%
Zhang et al.	NR	NR	NR
Miñan-Tapia et al.	NR	Adverse effects 11.7%	Acetaminophen (21.2%) Ibuprofen (10.8%) Dexamethasone (5.5%) Aspirin (4.4%) Azithromycin (1.2%)
Zavala-Flores et al.	NR	NR	Antibiotics (28.3%) Ivermectin (20.7%) Corticosteroids (17.0%) Acetaminophen (12.3%) Aspirin (4.7%) Hydroxychloroquine (0.9%) Others (1.9%)
Mansure et al.	NR	NR	NR

NR, Not Reported.

## 4 Discussion

Our study aimed to investigate the current scenario of self-medication during COVID-19 by systematically reviewing existing literature, according to the PRISMA statements [33,49]. Currently, there are articles in the literature on this subject conducted in various countries. The practices, knowledge, and attitude toward self-medication were assessed through questionnaires administered during cross-sectional studies.

Even though the studies were conducted in ten nations, there is a noticeable dearth of research in other regions, such as North America, Oceania, and North Africa. Self-medication trends are predicted to be influenced by differences in drug advertising, legislation, and the ability to obtain some medications without a prescription between countries [50,51]. Furthermore, twelve of the fourteen research that was considered took place in low to middle-income countries. Due to the health systems’ structure and condition, self-medication in these nations may be higher [52], and solid comparisons are impossible as a result of heterogeneity in assessments of self-medication between researchers.

Our study included fourteen investigations that evaluated the characteristics and prevalence of self-medication for COVID-19 management or prevention. Defining self-medication along with the recall timeframe was different across all the investigations. Rather than focusing on specified symptoms, most studies investigated self-medication in general: eleven in the general community (prevalence range: 19.3%–88.3%) and three in specified demographics (prevalence range: 33.9%–51.3%).

Self-medication is a worldwide practice that poses health concerns to individuals as well as communities [27,53]. Among 14 studies reviewed, the mean rate of self-medication prevalence during COVID-19 was estimated to be 44.78% around the world. Three systematic evaluations conducted in Ethiopia [33], Iran [31], and India [32] before COVID-19, found a prevalence rate of 44%, 53%, and 53.6%, respectively, with recall periods ranging from one day to six months. Similar estimates can be seen in these reports as well. A study, by Dare et al. reported that in Uganda, a significant decrease in the percentage of people practicing self-medication before the COVID-19 pandemic compared to the percentage of people practicing self-medication during the COVID-19 pandemic lockdown along with a decrease in the incidence of sickness during COVID-19 lockdown [35]. The decrease in the incidence of sickness might be a result of a change in the behavior of people to combat the spread of disease. During the lockdown, people paid more attention to their health as they were encouraged to practice more personal hygiene as washing of hands regularly and/or using sanitizers, wearing face masks, and maintaining social distance which might have resulted in the improvement of people's health status in the community. On the contrary, in an attempt to stop the spread of infection, most countries imposed lockdowns, curfews, border closures, and restricted movement from one district to another has resulted in low access to health care services.

Data from eight studies show that the most common causes to self-medicate were fever, respiratory problems, self-medication affordability, fear of stigma, and not believing the symptoms to be severe enough. At such a time of tumult, due to fear of COVID-19 and paucity of healthcare services for mild-moderate symptoms, people could have adapted to this new practice.

The preferred source of self-medication drugs was pharmacies. The study revealed that some over-the-counter pharmaceuticals namely non-steroidal anti-inflammatory drugs or acetaminophen were commonly used for the treatment of COVID-19 along with other diseases. But, the included studies found that medications (such as hydroxychloroquine or chloroquine, antibiotics, ivermectin, supplements or vitamins, and antiasthmatics) that are proven to have little or no benefit for the management or prevention of COVID-19 have a heterogeneous prevalence with self-medication according to COVID-19 guidelines set by Infectious Disease Society of America (IDSA) and WHO [54,55],

In reality, some of these treatments have serious side effects, such as antibiotic resistance generated by overuse of antibiotics, bleeding induced by aspirin, arrhythmia due to hydroxychloroquine, and suppression of the immune system that can be due to the use of corticosteroids [13]. Self-medication has been the subject of remarks from several healthcare organizations. For example, the World Health Organization understands that in many countries, successful self-medication may only be achieved by enhancing individuals' knowledge and education levels to the point where the potential harms may be evaded [16]. In the same way, many organizations support or promote proper non-prescription use of medications for example-the World Medical Association [56], the International Pharmaceutical Federation, and the World Self-Pharmaceutical Industry [57].

A study conducted on knowledge and self-medication with antibiotics in a Lebanese adult sample reported that self-medication was significantly associated with a low education level [58]. This result doesn't coincide with the present study conducted in the context of the COVID-19 outbreak. Indeed, participants with a secondary school level or higher were more likely to self-medicate. Self-medication was significantly associated with the level of education as those with degrees were more likely to self-medicate both before and during the outbreak [26]. These findings were similar in other studies as well and this has attributed to the vast knowledge of the medication as most medical graduates do possess a higher level of understanding of the over-the-counter drugs, along with their prescription and side effects [59]. In the present scenario, easy access to the internet and the ability to understand the information found on social media might be a reliable explanation for this trend [34].

The included papers in this study employed various forms of questions of the assessment self-medication, similar to what was discovered in prior systematic reviews [28,29], or they did not define the question utilized. Furthermore, different recall periods have been created in different studies. The studies assessing self-medication from the beginning of this pandemic showed contrasting periods based on the month they were conducted. Due to the lack of clarity, direct differentiation between studies or meta-analyses of their findings is prohibited [58], which might introduce bias into the internal validation of the results [60,61]. Furthermore, studies assessing self-medication using a pre-set listing of drugs may have produced skewed prevalence because these pharmaceuticals may not have included all of the most widely used medications [62].

As there is a clear limitation of primary studies regarding self-medication as evident from this review, more properly reported investigations, well-designed studies, and a uniform definition of self-medication are needed to fill this literature gap. Moreover, a meta-analysis was not performed which limits the statistical coherence and validity. Furthermore, because the COVID-19 pandemic is continuously evolving, additional research is needed to see if there is any difference regarding the scenario of self-medication between COVID-19 pandemic waves and after the implementation of major vaccines.

## 5 Conclusion

Based on findings, self-medication has been prevalent during this pandemic, along with the previous self-medication practices, COVID-19 has added a new trend to the existing burden. Self-medication has become a critical component of healthcare, but its provision is a huge global challenge, particularly in the wake of the COVID-19 epidemic. Self-medication has the potential to improve healthcare by lowering drug prescription prices. Inappropriate Self-medication, on the other hand, might result in a false diagnosis, significant side effects, drug interactions, drug habits, and germ resistance. As a result, there is a pressing need to regulate and supervise proper self-medication activities through strong laws and the involvement of healthcare professionals and policymakers. Similarly, extensive public health awareness programs on different platforms, proper monitoring of drug distribution, and legal action against medical malpractices should be strongly addressed. Educating general people about safe self-medication practices, hazards of superfluous drug usage, and provision of an affordable quality-health system will benefit everyone in the long run.

## Provenance and review

Not commissioned, externally peer-reviewed.

## Authors’ contributions

Conceptualization, methodology, and supervision: Abhigan Babu Shrestha, Sajina Shrestha; Resources: Abhigan Babu Shrestha, Fahmida Hoque Rimti, Data curation: Manjil Aryal, Junu Rana Magar, Labiba Hossainy; Writing original draft: All the authors participated equally; Editing: Fahmida Hoque Rimti; Correspondence: Fahmida Hoque Rimti.

## Sources of funding

N/A.

## Ethical approval

N/A.

## Consent

N/A.

## Registration of research studies

1. Name of the registry: PROSPERO
2. Unique Identifying number or registration ID: CRD42022312175
3. Hyperlink to your specific registration (must be publicly accessible and will be checked): **https://www.crd.york.ac.uk/prospero/display_record.php?RecordID=312175**
4. Name of the registry: Researchregistry.com
5. Unique Identifying number or registration ID: reviewregistry1414
6. Hyperlink to your specific registration (must be publicly accessible and will be checked): https://www.researchregistry.com/browse-the-registry#registryofsystematicreviewsmeta-analyses/registryofsystematicreviewsmeta-analysesdetails/62e29f7e8444c000242b1eb3/

## Guarantor

Fahmida Hoque Rimti.

## Declaration of competing interest

The authors have absolutely no conflicting interests.
