# Supplementary material for: The scenario of self-medication practices during the covid-19 pandemic; a systematic review
Source: Ann Med Surg (Lond). 2022 Aug 27;82:104482. doi: 10.1016/j.amsu.2022.104482 (PMC9419440; doi:10.1016/j.amsu.2022.104482)
Supplement: Multimedia component 2 [file mmc2.docx]

S1 Table. Search strategies.

**Scopus**

| ***Step*** | ***Queries*** | ***Findings*** |
| --- | --- | --- |
| 1 | ("COVID-19"/ "2019-nCoV" "coronavirus infection 2019" / "SARS-CoV- 2" / "severe acute respiratory syndrome coronavirus 2" / "severe acute respiratory syndrome corona virus 2" / "SARS-CoV2" /"SARS-CoV2" / "sars2" / / "new coronaviruses" / "ncov 2019" / "sars coronavirus 2") / (("coronavirus's"/"coronavirus" /"coronaviruses" /"new coronavirus" "coronaviruses" / "coronavirus-like" / "corona virus" / "corona viruses" / "ncov") / "cov" + ("outbreak" / / "2019 covid2019 / "COVID-19" / covid19 / 2019ncov / / "novel coronaviru*" / "2019 ncov"/"novel corona*"/ "new corona*"/ "novel coronaviruses" / "novel corona virus" / /  covid19 / "covid 19" / "sars cov 2" / sars2 ) | 109.348 |
| 2 | ("self medication" /"over-the-counter drug/pharmaceuticals*" / "nonprescription drug/pharmaceuticals*" / "non prescription drug/pharmaceuticals*" / "OTC drug/pharmaceuticals*" / "drug/pharmaceuticals hoarding" / "drug/pharmaceuticals use" /  "medication use" | 60,412 |
| 3 | 1 + 2 | 153 |

**Pubmed**

| ***Step*** | ***Queries*** | ***Findings*** |
| --- | --- | --- |
| 1 |  | 97,782 |
| 2 | ("self medication" /"over-the-counter drug/pharmaceuticals*" / "nonprescription drug/pharmaceuticals*" / "non prescription drug/pharmaceuticals*" / "OTC drug/pharmaceuticals*" /  "drug/pharmaceuticals hoarding" / "drug/pharmaceuticals use" / "medication use" | 45,070 |
| 3 | 1 + 2 | 121 |

**Embase**

| ***Step*** | ***Queries*** | ***Findings*** |
| --- | --- | --- |
| 1 | ("COVID-19"/ "2019-nCoV" "coronavirus infection 2019" / "SARS-CoV- 2" / "severe acute respiratory syndrome coronavirus 2" / "severe acute respiratory syndrome corona virus 2" / "SARS-CoV2" /"SARS-CoV2" / "sars2" / / "new coronaviruses" / "ncov 2019" / "sars coronavirus 2") / (("coronavirus's"/"coronavirus" /"coronaviruses" /"new coronavirus" "coronaviruses" / "coronavirus-like" / "corona virus" / "corona viruses" / "ncov") / "cov" + ("outbreak" / / "2019 covid2019 / "COVID-19" / covid19 / 2019ncov / / "novel coronaviru*" / "2019 ncov"/"novel corona*"/ "new corona*"/ "novel coronaviruses" / "novel corona virus" / /  covid19 / "covid 19" / "sars cov 2" / sars2 ) | 193,264 |
| 2 | ("self medication" /"over-the-counter drug/pharmaceuticals*" / "nonprescription drug/pharmaceuticals*" / "non prescription drug/pharmaceuticals*" / "OTC drug/pharmaceuticals*" / "drug/pharmaceuticals hoarding" / "drug/pharmaceuticals use" /  "medication use" | 15,712 |

| 3 | 1 + 2 | 148 |
| --- | --- | --- |

**Web of Science**

| ***No.*** | ***Queries*** | ***Findings*** |
| --- | --- | --- |
| 1 | ("COVID-19"/ "2019-nCoV" "coronavirus infection 2019" / "SARS-CoV- 2" / "severe acute respiratory syndrome coronavirus 2" / "severe acute respiratory syndrome corona virus 2" / "SARS-CoV2" /"SARS-CoV2" / "sars2" / / "new coronaviruses" / "ncov 2019" / "sars coronavirus 2") / (("coronavirus's"/"coronavirus" /"coronaviruses" /"new coronavirus" "coronaviruses" / "coronavirus-like" / "corona virus" / "corona viruses" / "ncov") / "cov" + ("outbreak" / / "2019 covid2019 / "COVID-19" / covid19  / 2019ncov / / "novel coronaviru*" / "2019 ncov"/"novel corona*"/ "new  corona*"/ "novel coronaviruses" / "novel corona virus" / / covid19 / "covid 19" / "sars cov 2" / sars2 ) | 126,378 |
| 2 | ("self medication" /"over-the-counter drug/pharmaceuticals*" / "nonprescription drug/pharmaceuticals*" / "non prescription drug/pharmaceuticals*" / "OTC drug/pharmaceuticals*" / "drug/pharmaceuticals hoarding" / "drug/pharmaceuticals use" /  "medication use" | 45,621 |
| 3 | 1 + 2 | 87 |

# MedRxiv

Website- <https://www.medrxiv.org/> bioRxiv and medRxiv

Find COVID-19 preprints Find:-

| ***No.*** | ***Query*** | ***Findings*** |
| --- | --- | --- |
| 1 | self-medication | 56 |
| 2 | Over the counter pharmaceuticals/drug/pharmaceuticals | 65 |
| 3 | Non-prescription pharmaceuticals/drug/pharmaceuticals | 28 |
| 4 | 1+2+3 | 149 |

# Scielo preprints

Website- <https://preprints.scielo.org/index.php/scielo/preprints> Find

| ***No.*** | ***Query*** | ***Findings*** |
| --- | --- | --- |
| 1 | Self-medication | 1 |

# Google

Enter- <https://www.google.com.pe/>

# Search:

First 100 papers reviewed

# Google Scholar

Enter- <https://scholar.google.com/>

**Search:** drug/pharmaceuticals use covid-19 self-medication
