# Supplementary material for: The scenario of self-medication practices during the covid-19 pandemic; a systematic review
Source: Ann Med Surg (Lond). 2022 Aug 27;82:104482. doi: 10.1016/j.amsu.2022.104482 (PMC9419440; doi:10.1016/j.amsu.2022.104482)
Supplement: Multimedia component 3 [file mmc3.docx]

**Quality assessment paper**

| Author | Score by ABS | Score by SS | Mean score | Inclusion |
| --- | --- | --- | --- | --- |
| Sadio et al | 8 | 8 | 8 | Include |
| Dare et al | 6 | 6 | 6 | Include |
| Rathi et al | 5 | 5 | 5 | Include |
| Chopra et al | 7 | 7 | 7 | Include |
| Azhar et al | 8 | 7 | 7.5 | Include |
| Quispe-Canari et al | 8 | 7 | 7.5 | Include |
| Heshmatifar et al | 6 | 5 | 6.5 | Include |
| Nasir et al | 5 | 5 | 5 | Include |
| Rafiq et al | 6 | 5 | 6.5 | Include |
| Wegbom et al | 7 | 8 | 7.5 | Include |
| Zhang et al | 7 | 7 | 7 | Include |
| Miñan-Tapia et al | 4 | 4 | 5 | Include |
| Zavala Flores et al | 4 | 4.5 | 4 | Include |
| Mansuri et al | 5 | 5 | 5 | Include |
