# Supplementary material for: The scenario of self-medication practices during the covid-19 pandemic; a systematic review
Source: Ann Med Surg (Lond). 2022 Aug 27;82:104482. doi: 10.1016/j.amsu.2022.104482 (PMC9419440; doi:10.1016/j.amsu.2022.104482)
Supplement: Multimedia component 4 [file mmc4.docx]

**The risk of bias tool proposed by the Joanna Briggs Institute for critical appraisal of cross-sectional studies**

| 1. Were the criteria for inclusion in the sample clearly defined? |
| --- |
| 1. Were the study subjects and the setting described in detail? |
| 1. Was the exposure measured in a valid and reliable way? |
| 1. Were objective, standard criteria used for measurement of the condition? |
| 1. Were confounding factors identified? |
| 1. Were strategies to deal with confounding factors stated? |
| 1. Were the outcomes measured in a valid and reliable way? |
| 1. Was appropriate statistical analysis used? |
